# Initial outcomes and safety of MR guided focused ultrasound (MRgFUS) thalamotomy for essential tremor of the first FUS Medical Unit in Portugal

**DOI:** 10.1055/s-0044-1788668

**Published:** 2024-08-26

**Authors:** Nuno Vila-Chã, Clara Chamadoira, Rui Araújo, Domingos Oliveira, Joana Alves Costa

**Affiliations:** 1 HIFU MRg Unit - JCC Diagnostic Imaging & HOPCA Saúde, installed in partnership at Hospital São Francisco do Porto, Portugal.

**Keywords:** Essential Tremor, Ultrasonic Therapy, Thalamus, Tremor Essencial, Terapia por Ultrassom, Tálamo

## Abstract

**Background** The magnetic resonance imaging-guided focused ultrasound (MRgFUS) has emerged as an innovative treatment for patients with medically refractory essential tremor (ET).

**Objective** This retrospective observational study aims to present the results of the first five patients with medically refractory ET who underwent MRgFUS treatment at this pioneering medical unit in Portugal.

**Methods** We conducted a retrospective chart review for the first five patients who underwent unilateral MRgFUS thalamotomy of the ventral intermediate (Vim) nucleus to treat medically refractory ET at our medical unit.

**Results** The mean patient age was 65.4 (26–84) years, and 60% were male. All patients had a family history of ET. The mean duration of disease was 17.4 years (range 10–24 years), and their dominant hand was the right. According to personal preference, the thalamotomy was performed on the left side in four patients, and on the right side in one. The MRgFUS thalamotomy led to significant improvements in both the clinical rating scale for tremor (CRST) score, by 62%, and the CRST composite score for the treated hand, by 73%. All patients experienced improvements in functionality and quality of life, by 52%. No severe adverse events were observed, and those that did occur during and following the procedure were mild and transient.

**Conclusion** The initial results from Portugal's first MRgFUS medical unit indicate promising outcomes, with improvement in quality of life, as well as mild and temporary adverse events These findings contribute to the growing body of literature supporting the efficacy and safety of MRgFUS as a viable treatment option for patients with medication-resistant ET.

## Resumo

**Antecedentes** A talomotomia por ultrassons focados - guiada por imagem de ressonância magnética (MRgFUS) surgiu recentemente como um tratamento inovador para pacientes com tremor essencial (TE) refratário ao tratamento médico.

**Objetivo** Este estudo observacional retrospectivo tem como objetivo apresentar os resultados dos primeiros cinco pacientes com TE refratário à medicação submetidos ao tratamento com MRgFUS numa unidade médica pioneira em Portugal.

**Métodos** Realizamos uma revisão retrospectiva dos dados clínicos dos cinco primeiros pacientes submetidos a talamotomia MRgFUS unilateral do núcleo ventral intermédio (Vim) para tratamento do TE medicamente refratário em nossa unidade médica.

**Resultados** A idade média dos pacientes era de 65,4 (26–84) anos, e 60% eram do sexo masculino. Todos os pacientes tinham história familiar de TE. A duração média da doença foi de 17,4 anos, e todos eram destros. De acordo com a preferência individual, em 4 pacientes, a talamotomia foi realizada no lado esquerdo, e em um, no lado direito. A talamotomia por MRgFUS levou a melhorias significativas tanto na pontuação da escala de classificação clínica para tremor (CRST), de 62%, e na pontuação composta da CRST para a mão tratada, de 73%. Todos os pacientes apresentaram melhorias na funcionalidade e na qualidade de vida, de 52%. Não foram observados efeitos adversos graves e os que ocorreram durante e após o procedimento foram ligeiros e transitórios.

**Conclusão** Os resultados iniciais da primeira unidade médica MRgFUS de Portugal indicam melhorias na qualidade de vida dos pacientes, com efeitos adversos ligeiros e transitórios. Estes dados reforçam a evidência crescente sobre a eficácia e segurança da talamotomia por MRgFUS como opção viável para pacientes com TE resistente à medicação.

## INTRODUCTION

Magnetic resonance imaging-guided focused ultrasound (MRgFUS) has gained considerable attention in recent years as a promising, non-invasive therapeutic modality for various medical conditions, including movements disorders, neuropathic pain, and neoplasm. 1 This innovative treatment offers the potential for precise tissue ablation with minimal damage to surrounding structures, making it an appealing option for patients who are unsuitable for or prefer to avoid invasive surgery. 2

Essential tremor (ET) is the most common movement disorder. Individuals frequently experience debilitating symptoms that substantially impair their quality of life. 3 4 Medications such as primidone and propranolol are first-line therapies, but a significant number of ET patients eventually become unresponsive to pharmacological treatments. 5

Recently, MRgFUS thalamotomy has emerged as an innovative treatment for patients with medically refractory ET. 6 Following the publication of a landmark randomized controlled trial that demonstrated the safety and efficacy of MRgFUS thalamotomy in ET, other centers have reported sustained long-term benefits from the procedure. 7 8 9 10 Despite the rapidly rising number of indications, this technology is still not widely available. Recently, the first medical unit offering MRgFUS treatments was established in Portugal, constituting an expansion of the current evidence base into a novel clinical and geographical context.

This retrospective observational study aims to present the results of the first five patients with medically refractory ET who underwent MRgFUS treatment at this pioneering medical unit in Portugal. We will discuss the clinical outcomes, safety profile, and improvements in functionality and quality of life associated with the procedure for these patients.

## METHODS

### Patients

We conducted a retrospective chart review for the first 5 patients who underwent unilateral MRgFUS thalamotomy of the ventral intermediate (Vim) nucleus to treat medically refractory ET at our FUS Medical Unit, using a 650-kHz system, Exablate Neuro 4000 Type 1.1 (INSIGHTEC Ltd., Tirat Carmel, Israel).

A neurologist specializing in movement disorders confirmed the ET diagnosis according to established criteria. 11 Refractory tremor was defined as disabling tremor persisting after at least two adequate treatment regimens. A Vim thalamotomy was performed on the opposite side of the patient's preferred hand.

All patients were eligible for the procedure, with no contraindications such as cognitive decline, neurodegenerative diseases, brain tumors, vascular malformations, severe unstable medical conditions, previous brain procedures, skull density ratio (SDR) of < 0.35, or contraindications for magnetic resonance imaging (MRI). Informed consent for the procedure was obtained as part of standard clinical care, in accordance with the Helsinki declaration. The requirement for additional consent was waived due to its nature as a retrospective study.

### Clinical procedures

A comprehensive chart review was conducted to collect demographic data (age, gender), disease characteristics (family history, duration, dominant hand), treatment parameters (SDR, skull area, number of high intensity focused ultrasound elements activated, sonication parameters including total number, mean and maximum temperature, and mean and maximum energy delivered), and follow-up information (tremor scores, functional status, quality of life, and adverse events).

Tremor severity was assessed using the clinical rating scale for tremor (CRST), which ranges from 0 to 160 points, with higher scores indicating greater disability. 12 Additionally, the treated hand's score was calculated separately with a composite tremor score (max score: 32; where higher scores indicate more severe tremor) derived from CRST part A (resting, postural, and action/intention components of upper extremity [UE] tremors) and part B (handwriting; drawing; A, B, and C; and pouring) in the hand contralateral to the thalamotomy.

The functional status was assessed using the CRST part C (max score: 32), with higher scores indicating more severe tremors. The score consists of 8 subscores, each evaluating various activities, such as speaking, eating, drinking, personal hygiene, dressing, writing, working, and social interactions.

Quality of life in ET patients was measured using the Quality of Life in Essential Tremor Questionnaire (QUEST), with scores ranging from 0 to 120, and higher ones indicating lower quality of life. 13 This is a patient self-report questionnaire designed to evaluate the impact of tremor symptoms on five distinct aspects of quality of life: communication, work and finances, hobbies and leisure activities, physical activities, and psychological wellbeing.

Adverse events were evaluated based on the Clavien-Dindo classification, which range from 1 to 5, with higher scores indicating more severe events. 14

These assessments were conducted at baseline, and at 1, 3, and 6 months, depending on each patient's follow-up duration. The most recent evaluation was used to calculate the tremor scores and QUEST outcomes.

### Imaging procedure

Preplanning brain computed tomography (CT) and MRI exams were performed on patients who were clinical candidates. The CT scan followed a special protocol with 1-mm slice thickness reconstructions and specific post-processing to determine skull density ratio (SDR), which is a limiting factor in successfully producing a thermal lesion. Furthermore, MRI exam also followed a specific protocol with high resolution T2 sagittal images, axial T2 fast spoiled gradient-echo (FSGRE) and fluid attenuated inversion recovery (FLAIR) images, isotropic (volumetric) sequences, namely 3D T2 Cube, 3D T1 MP-RAGE and 3D fast gray matter acquisition T1 inversion recovery (FGATIR), besides an axial diffusion-weighted imaging (DWI) with mesure of ADC map.

Patients with SDR > 0.35 and with no lesions that contraindicate the ablation procedure detected either by CT or MRI (e.g. brain tumors, acute ischemic lesion, and arterial or venous malformations) were considered confirmed candidates to the MRgFUS ablation procedure.

After MRgFUS, a control MRI scan was performed 12 to 24 hours after the procedure to evaluate the ablation lesion and its vasogenic oedema. The examination protocol included a volumetric 3D sagittal T1 MPRAGE with reconstructions in coronal and axial plans, axial T2 FSGRE and FLAIR with fat saturation (FS), as well as DWI (b50, b500, b1000) with ADC map.

### Surgical procedure

All procedures were performed in a SIGNA Architect AIR Edition (GE Medical Systems, Milwaukee, WI, USA) 3-T MRI system, using an Exhalate Neuro 4000 Type 1.1 (INSIGHTEC, Haifa, Israel). After head shaving, patients were placed in a stereotactic head frame under local anesthesia. A membrane was carefully attached to the head as low as possible to prevent air bubbles between it and the skin, coupled to an MRI-compatible ultrasound transducer using degassed water. Vim nucleus localization was defined at the level of the most posterior 1/4 of the anteromesial distance anterior to the posterior commissure (PC), and 14 mm lateral to the midline or 11.5 mm lateral the wall of the third ventricle in cases of ventriculomegaly. After real-time MRI targeting, acoustic energy was delivered with slowly increasing intensity to reach temperatures sufficient for tissue ablation (approximately 55–60° C).

Based on the tremor reduction, a submillimeter target modification was possible, and done in cases in which the tremor was not sufficiently reduced as expected according to the energy deliver with the first target verification sonication. After moving the target, verification with low energy test sonications was performed, followed by neurology assessment concerning tremor reduction and eventual adverse effects. Only after good response (tremor reduction without adverse effects) did sonication proceed to the treatment level, with higher energy delivery. Normally, two or three treatments were enough to produce the desired tremor reduction, finalizing the ablation itself. After the procedure, the transducer and head frame were removed. An MRI was performed 12 to 24 h after the procedure. Patients were discharged 24 h after the procedure. 6

### Statistical analysis

Continuous variables are presented as mean ± standard deviation (range), and categorical variables as frequency (%). Tremor score comparisons were conducted using a paired t-test. The threshold for statistical significance in group comparisons was set at *p* < 0.05. Percentage of changes in tremor and quality of life were calculated by subtracting pre- from posttreatment scores, then dividing the result by the pretreatment score, and multiplying by 100. Statistical analyses were performed using the SPSS Statistics for Windows, (IBM Corp., Armonk, NY, USA), version 25.0.

## RESULTS

### Demographics

A total of 5 cases were included in the study ( Table 1 ). The mean patient age was 65.4 ± 20.5 (26–84) years, and 60% were male. All patients had a positive family history. The mean duration of disease was 17.4 ± 5.6 (10–24) years, and the dominant hand for all patients was the right. According to the patients' personal preference, in 4 patients, the thalamotomy was performed on the left side, and in the remaining one, it was on the right side. Regarding follow-up durations, 2 patients reached 6 months, 1 patient had 3 months, and 2 patients had reached 1 month at the time of the study ( Table 1 ).

**Table 1:** Demographic, clinical characteristics and results

Patient	Age (years)	Gender	Family history	Disease duration (years)	Dominant hand	Laterality thalamotomy	Follow-up (months)	CRST total baseline	CRST total end follow-up	Improvement (%)
**1**	84	M	Yes	24	R	L	6	67	26	61
**2**	70	F	Yes	15	R	L	6	66	34	49
**3**	68	F	Yes	10	R	L	3	51	16	69
**4**	26	M	Yes	14	R	R	1	52	19	63
**5**	79	M	Yes	24	R	L	1	56	18	67
	(65.4 ± 20.5)			(17.4 ± 5.6)				(58.4 ± 6.8)	(22.6 ± 6.6)	62 ± 7% ( *p* < 0.05)

Abbreviations: F, female; M, male; R, right; L, left; CRST, Clinical Rating Scale for Tremor.

### Tremor scores, functionality, and quality of life

The MRgFUS thalamotomy led to significant improvements (reductions) in both the CRST score and composite score for the treated hand. The mean total score decreased by 62 ± 7% ( *p* < 0.05), going from 58.4 ± 6.8 at baseline to 22.6 ± 6.6 in the last evaluation ( Table 1 ). Additionally, the mean composite score for the treated hand decreased by 73 ± 10% ( *p* < 0.05), moving from 18.2 ± 3.7 at baseline to 5.4 ± 2.5 in the last evaluation.

All patients experienced improvements in functionality and quality of life. The mean CRST part C score dropped by 72 ± 9% ( *p* < 0.05) from 17.8 ± 4.7 at baseline to 4.8 ± 1.6 in the last evaluation, while the mean QUEST score declined by 52 ± 7% ( *p* < 0.05) from 39.6 ± 6.9 at baseline to 19.2 ± 4.2 in the last evaluation.

### Treatment parameters

The mean SDR was 0.60 ± 0.03 (0.56–0.64), the mean number of sonications performed per treatment was 7.8 ± 1.6 (6–10), the mean highest acoustic energy used was 11,019.8 ± 2,498.5 J (8,500–15,249), the mean number of high-temperature therapeutic sonications (> 55° C) was 2.2 ± 0.4 (2–3), and the mean number of low-temperature therapeutic ones (50–54° C) was 1.2 ± 0.4 (1–2), as shown in Table 2 .

**Table 2:** Treatment parameters

Patient	SDR	Sonications per treatment (n)	Highest acoustic energy used (J)	Low temperature therapeutic sonications (50–54°C)	High temperature therapeutic sonications (> 55°C)
**1**	0.64	8	9,372	1	2
**2**	0.58	10	12,628	2	3
**3**	0.56	6	8,500	1	2
**4**	0.64	9	9,350	1	2
**5**	0.58	6	15,249	1	2
	0.60 ± 0.03	7.8 ± 1.6	11,019.8 ± 2,498.5	1.2 ± 0.4	2.2 ± 0.4

Abbreviations: SDR, skull density ratio; J, Joules.

### Imaging results

**Figure 1:**
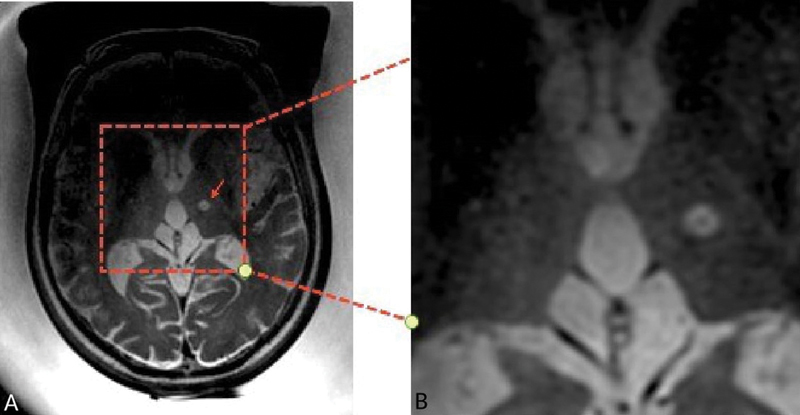
Intra MRgFUS ( **A** ) axial T2 fast spoiled gradient-echo (FSGRE) image and ( **B** ) detail acquired immediately after last sonication (patient still with the FUS helmet equipment positioned), showing a millimetric ablation lesion in the left thalamus on the Vim nucleus, with a very low signal intensity focus (dark spot) in the lesion core.

In Figure 1 , the dark spot represents the submillimeter hemosiderin deposition as a result of microhemorrhage in the center of the ablation lesion, which is expected in a successful treatment, proving that the lesion is consolidated.

**Figure 2:**
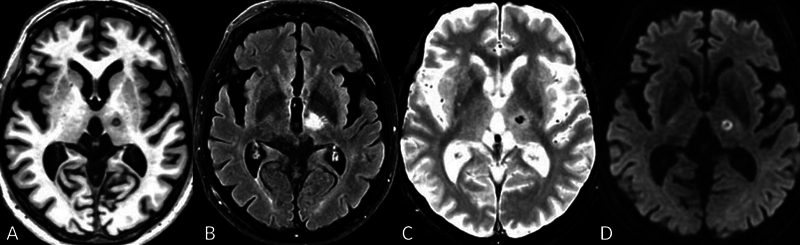
The MR axial images acquired 24 h after HIFU treatment ( **A** ) T1 weighted; ( **B** ) FLAIR; ( **C** ) T2*; and ( **D** ) DWI (B1000) images, showing the millimetric ablation lesion on the left Vim (A and D), the ablation lesion surrounded by vasogenic oedema (B) and the hemosiderin deposit with its blooming artifact highlighted by the susceptibility-weighted T2* sequence (C).

In Figure 2 , the blooming artifact grossly overestimates the size of the microhemorrhage, which is more realistically depicted on a DWI ( Figure 2D ), as the central dark spot on the high signal lesion. For a successful ablation ( Figure 2 A, B, C and D ), it is expected to create a submillimeter microhemorrhage ( Figure 2C ) on the core of the lesion, confirming it is a consolidated lesion. In Figure 2D , DWI B1000, the lesion itself appears with high signal by diffusion restriction, corresponding to the cytotoxic oedema which represents necrosis. If we compare the images in Figures 2B FLAIR, and 2D , it is easy to understand that most of the high signal seen on the FLAIR image does, indeed, correspond to the vasogenic oedema (transient) and not to the cytotoxic one (necrosis), as is latter shown on DWI image in Figure 2D .

### Adverse events

No severe adverse events were observed throughout the study period. All adverse events during and following the procedure were mild (Clavien-Dindo classification, grade 1) and transient. At the time of the last evaluation, no patients reported any side effects ( Table 3 ).

**Table 3:** Adverse events

Patient	Intervention	1 month	3 months	6 months
**1**	Nausea (1)	None	None	None
**2**	Nausea (1)	Tingling in the hand (1)	None	None
**3**	Dizziness (1)	Unsteady gait (1)	None	
**4**	Headache (1) Dizziness (1)	None		
**5**	Headache (1)	None		

## DISCUSSION

This study presents the results of the first five patients with medically refractory ET treated at the inaugural Portuguese MRgFUS medical unit. All patients experienced a significant improvement in tremor intensity, reaching a 73% reduction when considering the treated hand. This improvement is reflected in the enhanced functionality and quality of life for these patients. Our findings are consistent with the significant tremor reduction reported by Elias et al., 7 providing further evidence of the efficacy of MRgFUS thalamotomy, also observed in the 5-year follow-up study by Cosgrove et al. 3

Similar to the findings by Sinai et al. 9 and Lak et al., 10 our study observed only mild and transient adverse events. No serious or persistent adverse events, such as hematomas, occurred in any patients.

In our view, these results further substantiate the efficacy and safety of the MRgFUS Vim thalamotomy for refractory ET.

The major limitations of this study include its small sample size, limited follow-up duration, and the single-center design. Further research with a larger number of patients is necessary to assess the long-term outcomes at our center and to identify potential predictors of procedural success.

In conclusion, the initial results from Portugal's first MRgFUS medical unit, which involved five patients with refractory ET, indicate promising outcomes. Patients demonstrated improvements in their quality of life and experienced minimal adverse events. These findings contribute to the growing body of literature supporting the efficacy and safety of MRgFUS as a viable treatment option for patients with medication-resistant ETs. Furthermore, studies have demonstrate its effectiveness and safety, with no associated persistent adverse effects,which enables qualifying patients to have their second side treated at least 9 months after their first side treated. 15 Long-term follow-up of these patients is crucial in assessing the durability of MRgFUS treatment effects, identifying the potential late-onset side effects, and refining our understanding of optimal treatment parameters and patient selection criteria. It is likely that the indications for MRgFUS will continue to expand in the following years, such as tremor-dominant Parkinson disease 16 and motor complications of Parkinson disease. 17

In our experience, lack of awareness about MRgFUS and its indications among patients and referencing physicians is still a significant problem, and it is possible that many patients are not receiving the best possible treatment. Establishing Portugal's first MRgFUS medical unit represents a significant advancement in providing state-of-the-art, non-invasive therapeutic options to patients with ET, as well as and enriching the burgeoning global discourse with critical empirical data on MRgFUS thalamotomy.
